# Pharmacologically active microcarriers influence VEGF-A effects on mesenchymal stem cell survival

**DOI:** 10.1111/j.1582-4934.2012.01662.x

**Published:** 2013-01-11

**Authors:** Claudia Penna, Maria-Giulia Perrelli, Jean-Pierre Karam, Carmelina Angotti, Claudio Muscari, Claudia N Montero-Menei, Pasquale Pagliaro

**Affiliations:** a Department of Clinical and Biological Sciences, University of TurinTorino, Italy; b Department of Biochemistry G. Moruzzi, University of BolognaBologna, Italy; c Ingénierie de la Vectorisation Particulaire, LUNAM UniversitéAngers, France; d INSERM U1066, MINT “Bio-inspired Micro and Nanomedicine”Angers, France; e National Institute for Cardiovascular ResearchesBologna, Italy

**Keywords:** microspheres, drug release, growth factor, hypoxia, transplantation, stem cells

## Abstract

Resistance of transplanted mesenchymal stem cells (MSCs) in post-ischemic heart is limited by their poor vitality. Vascular-endothelial-growth-factor-A (VEGF-A) as such or slowly released by fibronectin-coated pharmacologically-active-microcarriers (FN-PAM-VEGF) could differently affect survival kinases and anti-apoptotic mediator (*e.g*. Bcl-2). Therefore VEGF-A or FN-PAM-VEGF could differently enhance cell proliferation, and/or resistance to hypoxia/reoxygenation (H/R) of MSCs. To test these hypotheses MSCs were incubated for 6-days with VEGF-A alone or with FN-PAM-VEGF. In addition, MSCs pre-treated for 24-hrs with VEGF-A or FN-PAM-VEGF were subsequently exposed to H/R (72-hrs 3% O_2_ and 3-hrs of reoxygenation). Cell-proliferation and post-hypoxic vitality were determined. Kinases were studied at 30-min., 1- and 3-days of treatment. Cell-proliferation increased about twofold (*P* < 0.01) 6-days after VEGF-A treatment, but by a lesser extent (55% increase) with FN-PAM-VEGF (*P* < 0.05). While MSC pre-treatment with VEGF-A confirmed cell-proliferation, pre-treatment with FN-PAM-VEGF protected MSCs against H/R. In the early phase of treatments, VEGF-A increased phospho-Akt, phospho-ERK-1/2 and phospho-PKCε compared to the untreated cells or FN-PAM-VEGF. Afterword, kinase phosphorylations were higher with VGEF, except for ERK-1/2, which was similarly increased by both treatments at 3 days. Only FN-PAM-VEGF significantly increased Bcl-2 levels. After H/R, lactate dehydrogenase release and cleaved Caspase-3 levels were mainly reduced by FN-PAM-VEGF. While VEGF-A enhances MSC proliferation in normoxia, FN-PAM-VEGF mainly hampers post-hypoxic MSC death. These different effects underscore the necessity of approaches suited to the various conditions. The use of FN-PAM-VEGF could be considered as a novel approach for enhancing MSC survival and regeneration in hostile environment of post-ischemic tissues.

## Introduction

In spite of the beneficial effects observed in cell therapy after myocardial infarction, retention, survival and functionality of transplanted cells still need to be improved. Many investigators attempted to modify the ratio ‘organ tissue damage/repair’ by means of stem cell-based regenerative therapies during the last decade [1, 2]. Results from recent clinical studies with infarcted patients showed improved cardiac function following injection of adult stem cells from different sources including bone marrow and peripheral blood [2–4]. Mesenchymal stem cell (MSCs) transplantation is a promising strategy. However, cell replacement therapy is limited by the poor vitality of transplanted MSCs, especially in hypoxic environment [5, 6].

It has been reported that the capacity of transplanted bone marrow MSCs to survive and subsequently integrate into host heart may be so poor that about 99% of transplanted cells are lost during the first 24-hrs after transplantation [6, 7]. In fact, after infusion or injection into an ischemic tissue, MSCs face a hostile, inflammatory environment that may strongly limit their function and survival. Another important process to be considered during the implantation of MSCs in the injured organ is the formation of new vasculature. In fact, it is necessary that during the repair of the ischemic tissue an appropriate angiogenesis occurs [8].

Experimental observations have shown the plasticity of bone marrow MSCs indicating that in appropriate conditions these cells can repair a damaged tissue [2, 9]. The influence of endogenous factors such as cytokines, growth factors, and the local cellular milieu upon MSCs remains poorly understood [10–17]. Members of the vascular endothelial growth factor (VEGF) family and their receptors (VEGFR) play important roles in the development and maintenance of the blood and lymphatic vasculature. To date, five VEGFs have been identified in the mammalian (VEGF-A, -B, -C, -D, and placental growth factor), which display distinct binding affinities for VEGFR-1, -2, and -3. Several isoforms of VEGF-A (mainly VEGF_121_, VEGF_165_ and VEGF_189_) have been shown to display angiogenic properties [18]. In particular, in the brain, vessel formation after VEGF-A exposure has been observed already after 4 days [19].

Besides being a promoter of angiogenesis, VEGF-A, is considered a multifunctional growth factor; in particular, it promotes myocardial protection in the short term by decreasing cardiomyocyte apoptotic signaling and in the long term by increasing neovascularization and tissue perfusion [20, 21]. Bone marrow MSCs themselves are capable of producing VEGFs both in normo- and in hypoxic-conditions [22]. However, evidently, this production is not enough to promote their protection when transplanted [5–7]. Exogenous VEGF-A can directly augment myocardial function during acute ischemia/reperfusion [23]. Moreover, VEGF-A can induce the activation of survival protein kinases including Akt pathway, as well as the activation of antiapoptotic and growth/hypertrophy signaling pathways [24, 25]. Accordingly, one would expect that VEGF-A may increase the progeny formation of MSCs. However, the size of progenitor-cell compartment is governed by the balance between the cell gain (self renewal) and the cell loss (apoptosis, necrosis).

Therefore, an imbalance in renewal and apoptosis will result in an elevation or a fall in the progenitor cell mass [26]. It can be expected that the limitation of cell death in a hostile environment may require a prolonged activity of the protective agent. Since VEGF-A may affect both self renewal and apoptosis, and since pharmacologically-active-microcarriers (fibronectin-coated poly(lactic-co-glycolic acid (PLGA) microcarriers containing VEGF-A; FN-PAM-VEGF) have been developed, which allow *in situ* prolonged/controlled VEGF-A delivery and cell adhesion by their fibronectin biomimetic surface, we hypothesize that there are substantial differences on MSC survival depending on whether this growth factor is applied as a free compound or incorporated within FN-PAMs for a sustained release. Moreover, it is important to note that this approach combines the effect of the controlled release of VEGF-A to the 3D biomimetic support provided by the FN-PAMs. Indeed, it has been shown that FN and a 3D support together or separately stimulate survival of MSCs [27].

This would be consistent with studies which already used gelatin, PLGA and alginate microspheres as a biocompatible polymeric support for the controlled release of bioactive VEGF-A *in vivo* [27–29].

In the present study we compared *in vitro* whether or not FN-PAMs delivering VEGF_165_ (FN-PAM-VEGF) induce a different protective/proliferative effects compared to VEGF_165_ alone. In particular, we tested whether MSC survival in hypoxic conditions is differently influenced by FN-PAM-VEGF and VEGF_165_ pre-treatment.

## Materials and methods

Unless otherwise specified, reagents were purchased from Sigma-Aldrich (St. Louis, MO, USA). Tissue culture plasticware was obtained from M-Medical (Milan, Italy).

### Formulation of PAMs Releasing VEGF_165_

Poly(lactic-co-glycolic acid) (PLGA)-microspheres of an average diameter of 60 μm were prepared using an emulsion solvent extraction-evaporation process previously described [28]. The PLGA-copolymer is with a lactic:glycolic ratio of 37.5:25 (MW: 25,000 D; Phusis, Saint Ismier, France). The total protein loading was 0.6% w/w with respect to the amount of polymer, *i.e*. 0.1% VEGF_165_ and 0.5% HSA.

The used VEGF_165_ is a recombinant VEGF-A (Peprotech, Neuilly-Sur-Seine, France), and to simplify the reading, hereafter we referred to VEGF-A when it is used alone and to VEGF when it is used in composed words. First, NaCl and glycofurol, a water-miscible non-solvent of proteins, were used to precipitate the proteins separately, as previously described [30] and adapted to each protein. For VEGF-A a NaCl solution at 0.01 M at 4°C, containing a protein-poloxamer 188 excipient in an additive to protein ratio of 20:1 was added and mixed to glycofurol to form a 1 ml suspension. The same procedure was used for HSA with a NaCl solution at 0.3 M. Thirty minutes later, the protein nanoparticles were recovered by centrifugation (10,000 g, 30-min.). They were then carefully dispersed in the organic solution (667 μl; 3:1 methylene chloride:acetone) containing 50 mg polymer and emulsified in a poly (vinyl alcohol) aqueous solution (90 ml, 4% w/v) maintained at 1°C and mechanically stirred for 1-min. (Heidolph RZR2041, Merck Eurolab, Paris, France). After addition of 33 ml of deionized water and stirring for 10-min., the resulting o/w emulsion was added to deionized water (167 ml) and stirred at 550 r.p.m. further for 20-min. to extract the organic solvent. Finally, the formed microparticles were filtered on a 5 μm filter (HVLP type, Millipore SA, Guyancourt, France), washed with 500 ml of deionized water and freeze-dried. Unloaded microparticles were prepared in the same manner without adding the protein.

The microspheres were coated with 12 μg/ml of poly-D-lysine and 18 μg/ml of fibronectin (Sigma-Aldrich, Saint-Quentin Fallavier, France) as previously describes [31]. Briefly, microspheres were placed under rotation with the fibronectin-poly-D-lysine mixture for 90-min. at 15 r.p.m. in an incubator and were then freeze-dried and stored at 4°C for the experiments or immediately used for release kinetics studies. These fibronectin-coated microspheres are named FN-PAMs.

### Protein encapsulation efficiency

Protein encapsulation yield was determined considering both the VEGF-A biologically active entrapped protein and the total protein. Protein PLGA microspheres (5 mg) were dissolved in 1 ml acetone in silanized glass tube for 1 hr, the entrapped protein was separated from the dissolved polymer by centrifugation (15-min., 14,662 g) and the acetone was removed. To ensure that the entire polymer was dissolved, 1 ml of acetone was added and the solution was left to stand for one more hour, and then totally removed after centrifugation (15-min., 14,662 g). The pellet was resuspended in PBS. The encapsulation efficiency was measured using a protein dosage kit, NanoOrange test (Invitrogen, Cergy-Pontoise, France) and VEGF ELISA kits (Peprotech).

### Release kinetics, *in vitro* study

The *in vitro* release profile of protein from PLGA microspheres was determined by adding 250 μl of PBS buffer, pH 7.4, containing 1% w/v BSA to 2.5 mg of FN-PAM-VEGF into eppendorf tubes. The tubes were closed and incubated in a shaking water bath (37°C, 125 r.p.m.). The tubes were centrifuged for 5-min. at 664 g and 250 μl of the supernatant were collected for analysis and replaced by fresh buffer. This procedure was repeated at different time-points (1, 2, 3, 4, 7, 10, 14 and 21 days) and the released VEGF-A present in the collected aliquots was measured by ELISA (Peprotech). The theoretical amount of VEGF-A present in the 2.5 mg FN-PAM-VEGF was corrected using the results of the encapsulation yield allowing the actual amount of VEGF-A contained in the FN-PAM-VEGF to be established. The cumulative release of VEGF-A over time was then calculated.

To confirm the biological activity of the released VEGF-A a bioassay was performed using a human umbilical vein endothelial cells (HUVECs; Lonza, Warkerville, MD, USA) proliferation assay; HUVECs were cultured and passaged in standard endothelial cell medium (EGM-2 medium; Lonza) which includes a supplement of growth factors. The HUVECs were then plated (5 × 10^3^ cells) onto 24 well plates, with filtered medium (supplement-free). On these HUVECs proliferation was first investigated by the Alamar Blue assay (Invitrogen) using different concentrations of free VEGF-A at 24-hrs, 48-hrs, 5-days and 1-week time-points; the best effect was observed with 4 ng/ml VEGF-A for 5-days. Over a period of 5-days HUVEC proliferation was then investigated using the samples collected from FN-PAM-VEGF under the same conditions performed to evaluate the release kinetics of VEGF-A and at the time-points above reported. Each sample was diluted to 4 ng/ml of released VEGF-A, according to the ELISA results, and compared to the supplement-free medium alone or supplement-free medium containing 4 ng/ml of free VEGF-A.

### MSC isolation and cell culture

Mesenchymal stem cells were extracted from bone marrow of femurs of Wistar rats 6–12 months of age (weight 450–550 g; Janvier, Le Genest St Isle, France). MSCs were extracted by inserting a 21-gauge needle into the shaft of the bone and flushing with a solution of minimum essential medium eagle α (α-MEM) and 20% fetal bovine serum (FBS) (Sigma-Aldrich, Milan, Italy) implemented with 2 mM glutamine, 100 U/ml penicillin and 100 mg/ml streptomycin (Lonza); the cell suspension was filtered and cultured at 37°C. After 24-hrs the medium was replaced with α-MEM containing 10% FBS, 2 mM glutamine, 100 U/ml penicillin and 100 mg/ml streptomycin. We allowed MSCs to grow up to passage 3 (P3), replacing the medium every 2–3 days as reported in the literature [32–35].

To verify that the cell population we used for cultures was composed of MSCs, we showed that the cells were CD90 positive and CD34/CD45 negative [32–34]. Moreover, previous differentiation experiments performed in our laboratories showed the MSC potential to differentiate into adipocytes [34], osteoblasts and muscle cells [32]. The MSCs were then included in the study and used accordingly to the protocols described below.

Rats were used in accordance with the Italian law (DL-116, 27 January 1992) and the Guide for the Care and Use of Laboratory Animals published by the US National Institutes of Health (NIH Publication no. 85-23, revised 1996). The project was approved by the Italian Ministry of Health, Rome, and by the ethics review board of the University of Turin. Rats were anesthetized by i.p. injection of urethane (1 g/kg) and killed by decapitation [35].

### MSC adhesion to microspheres

FN-PAMs and FN-PAM-VEGF were used according to the following protocol: 0.5 ml of α-MEM with 10% FBS was added into the eppendorf containing the microspheres (0.5 mg) and incubate for 15-min. in order to resuspend them. Just before cell attachment, the solution containing microspheres was vortexed, put in an ultrasound bath for 30-sec. and vortexed again. Pilot experiments confirmed that this procedure avoids possible FN-PAM-aggregates; thus providing the largest surface for MSC adhesion, without affecting microsphere integrity. Then the microsphere suspension was put into ultra low attachment cluster plates (Corning, Sigma-Aldrich, Milano, Italy) and 9.0 × 10^4^ MSCs in 0.5 ml of culture medium were added.

### Normoxic and hypoxic experimental conditions

**(1)** Normoxia: *i.e. standard conditions* (normoxic: 21% O_2_ and 5% CO_2_) to study cell survival; these gas concentrations are identical to those used during MSC culture from isolation to P3.

**(2)** Hypoxia/reoxygenation (H/R): *i.e*. hypoxic mixture (3% O_2_ and 5% CO_2_) for 72-hrs and subsequent reoxygenation (21% O_2_ and 5% CO_2_) for 3-hrs to study cell survival.

#### Study of viability in standard-normoxia with and without treatment with VEGF-A, FN-PAMs or FN-PAM-VEGF

**Fig. 1:**
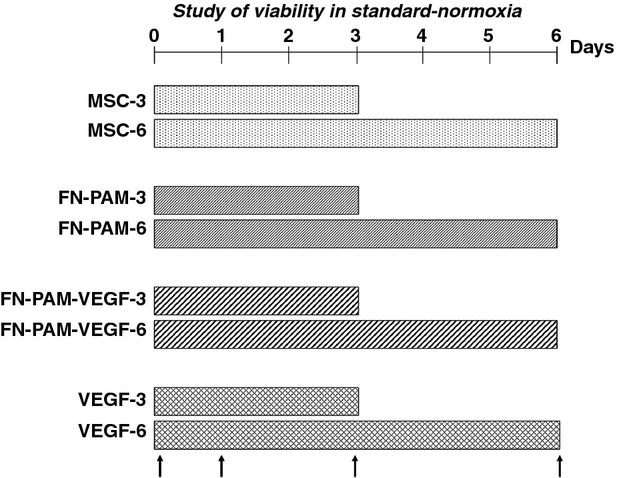
Time-lines and protocols for experimental groups in normoxia. Timing of various interventions is shown in relation to the onset of cell culture. Mesenchymal stem cells (MSCs) were kept under standard-normoxia conditions for 3- or 6-days (MSC-3 or MSC-6). Fibronectin-coated pharmacologically-active-microcarriers (FN-PAM), FN-PAM incorporating VEGF-A (FN-PAM-VEGF) or free VEGF-A were added at time 0, and after 3-days for the 6-days groups. Assessments were performed after 30-min. from the beginning of treatments and then 1-, 3- and 6-days after (upwards arrows). For other acronyms see also the text.

We carried out experiments with MSCs exposed to agents for 3 or 6 days (Fig. 1). Therefore the groups considered in this study were the following:

Control: MSCs were kept under standard conditions for 3-days (MSC-3) or 6 days (MSC-6);

FN-PAMs: 0.5 mg FN-PAMs were added to culture medium together with 9.0 × 10^4^ MSCs and kept under standard conditions for 3-days (FN-PAM-3) or 6-days (FN-PAM-6);

FN-PAM-VEGF: 0.5 mg VEGF/FN-PAM were added to culture medium together with 9.0 × 10^4^ MSCs and kept under standard conditions for 3-days (FN- PAM-VEGF-3) or 6-days (FN-PAM-VEGF-6);

VEGF-A: 9.0 × 10^4^ MSCs were cultured and kept under standard conditions, and then VEGF-A (9 ng) was added to the cell culture for 3-days (VEGF-3) or 6-days (N-VEGF-6).

#### Study of survival after H/R with and without pre-treatment with VEGF-A, FN-PAMs or FN-PAM-VEGF

**Fig. 2:**
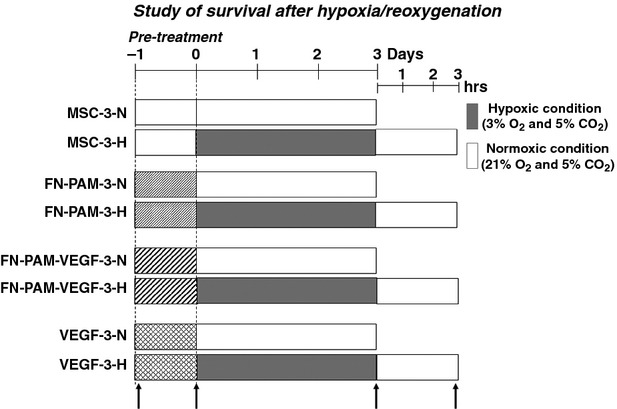
Time-lines and protocols for experimental groups in hypoxia/reoxygenation. Timing of various interventions is shown in relation to the onset of cell culture. Mesenchymal stem cells (MSCs) were kept under standard conditions for 1-day, then they were subjected to 3-days hypoxia and 3-hrs reoxygenation. Fibronectin-coated pharmacologically-active-microcarriers (FN-PAM), FN-PAM incorporating VEGF-A (FN-PAM-VEGF) or free VEGF-A were added at time 0. For comparative purpose we considered normoxic (-N) and hypoxic (-H) protocols. Assessments were performed after 30-min. from the beginning of treatments and then before and after hypoxia, and at the end of reoxygenation (upwards arrows). For other acronyms see also the text.

In this protocol, experiments were carried out with MSCs alone or MSC pretreated with the studied factors (FN-PAMs, FN-PAM-VEGF or VEGF-A; Fig. 2). In particular, the MSCs were pre-treated with VEGF-A (9 ng), FN-PAMs (0.5 mg) or FN-PAM-VEGF (0.5 mg) for 24-hrs. Before exposure to hypoxic condition, MSCs were incubated with trypsin-EDTA 0.25% solution and subjected to mild centrifugations (250–300 g for 5-min.) in order to separate cells from FN-PAMs or FN-PAM-VEGF. Then the medium was changed and cells were cultured in 2% FBS without treatment factors. Thereafter, subgroups of cells were subjected to a protocol of hypoxia/reoxygenation (72/3 hrs) in a hypoxic chamber (INVIVO_2_ 200, Belsar, Varese, Italy). Therefore the groups considered in the study of cell survival were the following:

Untreated MSCs kept under standard conditions for 3-days (MSC-3-N), and untreated MSCs exposed to H/R (MSC-3-H);

MSCs pre-treated with FN-PAM and kept under standard conditions (FN-PAM-3-N), and MSCs pre-treated with FN-PAMs and exposed to H/R (FN-PAM-3-H);

MSCs pre-treated with FN-PAM-VEGF and kept under standard conditions (FN-PAM-VEGF-3-N), and MSCs pre-treated with FN-PAM-VEGF and exposed to H/R (FN-PAM-VEGF-3-H);

MSCs pre-treated with VEGF-A and kept under standard conditions (VEGF-3-N), and MSCs pre-treated with VEGF-A and exposed to H/R (VEGF-3-H).

In brief, in each of the above four groups, for comparative purpose we considered normoxic (-N) and hypoxic (-H) protocols.

### Cell survival

At the end of experiments cell survival was assessed using the cell viability test 3-(4,5-Dimethylthiazol-2-yl)-2,5-diphenyltetrazolium bromide (MTT) assay (Sigma-Aldrich, Italy) [36]. The release of lactate dehydrogenase (LDH) was also checked at the beginning of the experiments and after 3- or 6-days using a dedicated assay (Sigma-Aldrich, Italy) [37].

### Western blotting

In order to compare the effects of FN-PAM-VEGF and VEGF-A on critical proteins for cell survival and apoptosis, the analysis of western blotting was performed on MSCs exposed to normoxic conditions after 30-min., 1- and 3-days of treatment; the analysis was also performed after exposure of MSCs to H/R conditions (see upward arrows of Figs 1 and 2).

About 50 μg of protein extracts were separated by SDS-10% PAGE and transferred to PVDF membranes (GE Healthcare, VWR, Milan, Italy). The membranes were incubated overnight with the following antibodies: anti-Akt (Cell Signaling, Euroclone, Pero (MI), Italy), anti-PKCε (Cell Signaling), anti-ERK1/2 (Cell Signaling), anti-Bcl-2 (Cell Signaling) and anti-cleaved Caspase-3 (Cell Signaling), and phosphorylated forms anti-phospho-Akt (Ser 473, Cell Signaling), anti-phospho-PKCε (Ser 729, Upstate, Prodotti Gianni, Milan, Italy) and anti-phospho-ERK1/2 (Thr 402-Tyr 204, Cell Signaling). All antibodies were diluted according to manufacturer's instructions. Western blotting analysis was displayed by the Immuno-Star HRP substrate (BioRad, Segrate (MI), Italy) and quantified by Kodak Image Station 440CF. The quantification of protein used was performed according to Bradford method [38]. The image analysis was performed using the Kodak 1D 3.5 software [39].

To confirm that equal amounts of protein were loaded membranes were incubated with anti-β actin (Sigma-Aldrich, Italy). For each condition, we normalized the expression of kinases to its matched loading control β actin and then to the mean values of MSC-3.

### Statistical analysis

Data were expressed as mean ± SEM and reported as percentage of control. The values were analyzed using anova and Newman-Keuls Multiple Comparison Test as post-anova test, and were considered significant for *P* < 0.05.

## Results

### VEGF-A encapsulation and release kinetics from FN-PAM-VEGF

**Fig. 3:**
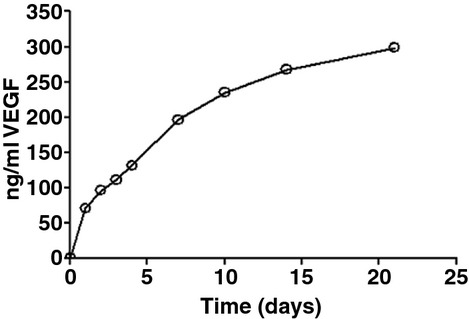
Illustrative release kinetics of VEGF-A from fibronectin-coated pharmacologically-active-microcarriers complexed with VEGF-A (FN-PAM-VEGF) and bioassay. The cumulative release of VEGF-A from FN-PAM up to 4 weeks was 21% that represent about 300 ng/ml of the entrapped protein. Each point represents the mean of triplicate experiments. The release profile was performed with 2.5 mg PAMs. VEGF-A collected from each sample of the release kinetics assay was able to stimulate HUVEC proliferation as an equal amount of native VEGF-A for a period of 7 days (data not shown).

The encapsulation yield of total protein into FN-PAM-VEGF was 67.8% and was 59.4% of biologically active VEGF-A. A nice continuous release of VEGF-A from these PAMs was observed for at least 3 weeks consisting in a cumulative release of 300 ng/ml for 2.5 mg FN-PAM-VEGF and representing 21% of the entrapped protein (Fig. 3). The VEGF-A collected from each sample of the kinetic release assay (diluted to 4 ng/ml) was able to stimulate HUVEC proliferation for a period of 5-days in a similar manner as the free VEGF-A at 4 ng/ml (data not shown). The bioassay performed with the HUVEC cells confirmed that the VEGF-A was released under a bioactive conformation during the entire period. One milligram FN-PAM was loaded with 1.0 μg VEGF-A. Based on the release kinetics study of VEGF-A, FN-PAM-VEGF released 17.8 ng VEGF-A/mg FN-PAM/day during the first week. Since we used 0.5 mg of FN-PAM-VEGF, the dose of VEGF-A was about 9 ng/day. Therefore, the doses of VEGF-A employed in the two conditions with the cells (FN-PAM-VEGF or VEGF-A only) are identical. This concentration of VEGF-A is similar (nanomolar range) to that used in other cellular models (explants of cerebral cortex) [40] and in the MSC *in vitro* [41].

### Cell proliferation analysis in normoxic conditions

**Fig. 4:**
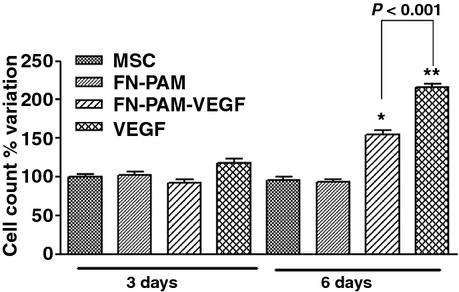
Cell growth in normoxia with and without factors (free-VEGF-A, FN-PAMs or FN-PAM-VEGF) after 3 and 6 days. Data are% variation with respect to mean value of MSCs kept under standard conditions for 3-days (MSC-3). After 3-days treatment there are not statistical differences among groups. After 6-days treatment cell growth was significantly increased by FN-PAM-VEGF-6 and even more by VEGF-6. *P < 0.05 *versus* other groups; **P < 0.001 *versus* all other groups. *n* = 5 for each condition.

In Figure 4, data are presented as percent variation with respect to mean value of cell count in control (MSC-3)**.** We found that there were no significant differences in cell viability between MSC and FN-PAM groups both after 3 and 6 days.

The treatment for 3-days in FN-PAM-VEGF-3 and VEGF-3 groups did not induce significant variations in cell viability with respect to MSC-3. However after 6-days, both VEGF-6 and FN-PAM-VEGF-6 increased significantly cell viability with respect to all other conditions (*P* < 0.01 and *P* < 0.05, respectively; Fig. 4). In particular, compared to control cell numbers were increased by FN-PAM-VEGF-6 and even more by VEGF-6 (+155 ± 6% and +216 ± 4%, respectively).

**Fig. 5:**
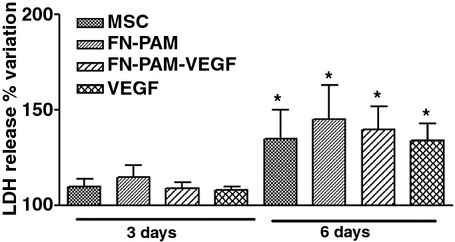
Lactate dehydrogenase (LDH) release. Data are% variation with respect to mean value observed in MSCs at the beginning of the experiments. LDH release increased similarly throughout the experiments in all groups. In fact, after 3 and 6 days treatments there are not statistical differences among groups. *P < 0.01 *versus* baseline.

While after 3-days LDH increase was not significant with respect to baseline level, it was significantly increased after 6-days treatment (Fig. 5). Since similar LDH release occurred among all groups after 3- and 6-days, *i.e*. no difference in cell death was observed throughout the experiments, the differences in cell number (Fig. 4) are likely due to different cell proliferation. These results suggest that *VEGF-A* has a more potent proliferative effect than FN-PAM-VEGF after 6 days of treatment in normoxia.

### Survival analysis after H/R in pretreated cells

**Fig. 6:**
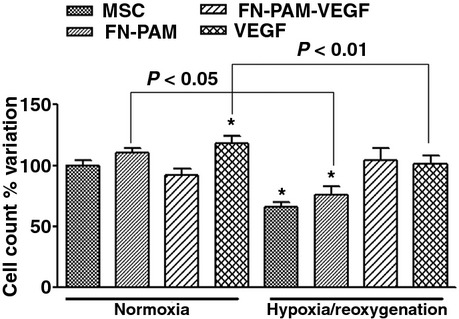
Cell growth in normoxia for 3-days (first four bars), and cell survival after 3-days hypoxia and 3-hrs reoxigenation (H/R), of MSC pre-treated or not with factors (free-VEGF-A, FN-PAMs or FN-PAM-VEGF) for 24-hrs. Data are% variation with respect to mean value of MSCs kept under standard conditions for 3-days (MSC-3-N). In normoxia only free-VEGF-A pre-treatment induced a significant increase in cell number. Only pre-treatment with FN-PAM-VEGF was able to counteract hypoxia-induced cell number reduction. *P < 0.01 *versus* MSC-3-N. *n* = 5 for each condition.

In Figure 6, data are presented as percent variation with respect to mean value of cell count in control conditions (MSC-3-N). The analysis of viability in normoxia showed that 24-hrs pre-treatment with either FN-PAMs or FN-PAM-VEGF did not influence cell numbers; however, VEGF-A pretreatment (VEGF-3-N) increased cell number. These data support the idea that free-*VEGF-A* has a major pro-proliferative effect.

In the absence of pre-treatment, the H/R protocol induced a 34% reduction in MSC number (*P* < 0.01, MSC-3-N *versus* MSC-3-H). Also in MSCs pretreated with empty FN-PAMs a similar percent reduction (−27%) of cells was observed (*P* < 0.05, FN-PAM-3-N *versus* FN-PAM-3-H). However the pre-treatment with FN-PAM-VEGF preserved cell survival and was able to counteract hypoxia effects (*P* = not significant between FN-PAM-VEGF-3-N and FN-PAM-VEGF-3-H), yet pre-treatment with free VEGF-A was not able to preserve survival; in fact a 38% reduction in MSC number was observed in VEGF-3-H (*P* < 0.01 *versus* VEGF-3-N). LDH data corroborated these results (data not shown).

These results suggest that pre-treatment with *FN-PAM-VEGF* limits cell mortality after H/R.

### Western Blot analysis

#### Analysis in normoxic conditions

Figures 9 show the total and phospho-kinase bands (central panels) with relative normalized ratio (left panels) and mean values of normalized phospho-enzyme levels (right panels), assessed after 30-min., 1- and 3-days of treatment, respectively. In these figures (A) MSC control group, (B) MSC treated with FN-PAM-VEGF, and (C) MSC treated with free-VEGF-A.

**Fig. 7:**
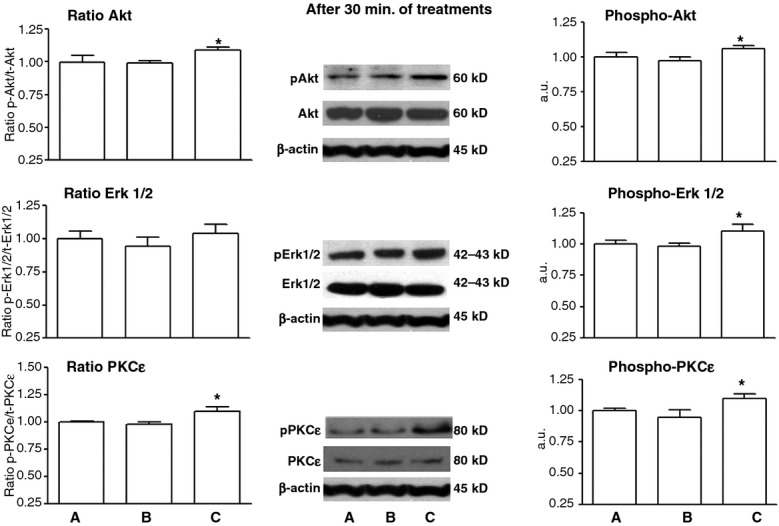
Western blot analysis of Akt (top panels), ERK1/2 (middle panels), and PKCε (bottom panels) after 30-min. (**A**) MSC control group (**B**) MSC treated with FN-PAM-VEGF, and (**C**) MSC treated with free-VEGF-A. The panels on the left are normalized phospho/total kinase ratios. The central panels are representative bands of total and phospho-kinases. The panels on the right show the normalized mean values of phospho-enzyme only. We normalized the expression of total kinases and phospho-kinases for each condition to its matched loading control β actin and then where normalized with respect to the mean values of MSCs kept under standard conditions for 30-min. After 30-min. treatment, only free-VEGF-A induces an increase in phosphorylation of Akt, ERK1/2 and PKCε. *P < 0.05 respect to MSC control group; *n* = 3 for each condition.

**Fig. 8:**
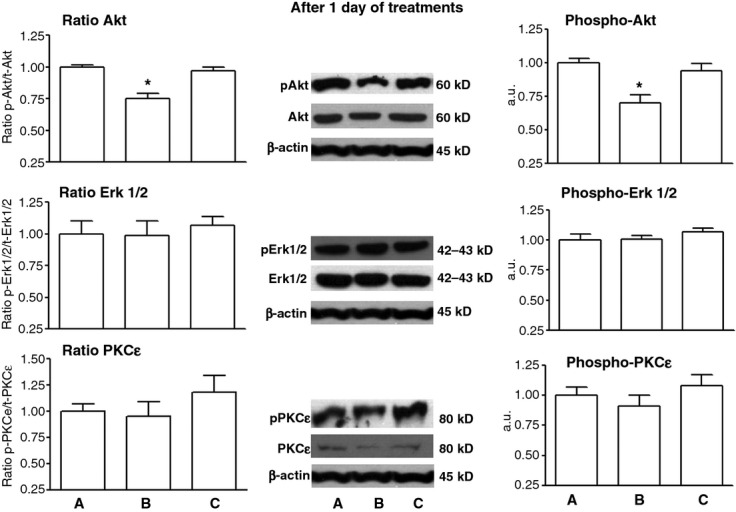
Western blot analysis of Akt (top panels), ERK1/2 (middle panels), and PKCε (bottom panels) after 1-day. (**A**) MSC control group (**B**) MSC treated with FN-PAM-VEGF, and (**C**) MSC treated with free-VEGF-A. The panels on the left are normalized phospho/total kinase ratios. The central panels are representative bands of total and phospho-kinases. The panels on the right show the normalized mean values of phospho-enzyme only. We normalized the expression of total kinases and phospho-kinases for each condition to its matched loading control β actin and then where normalized with respect to the mean values of MSCs kept under standard conditions for 1-day. Only FN-PAM-VEGF induces a significant reduction in phospho/total Akt ratio and phospho Akt level. *P < 0.05 respect to MSC control group; *n* = 3 for each condition.

**Fig. 9:**
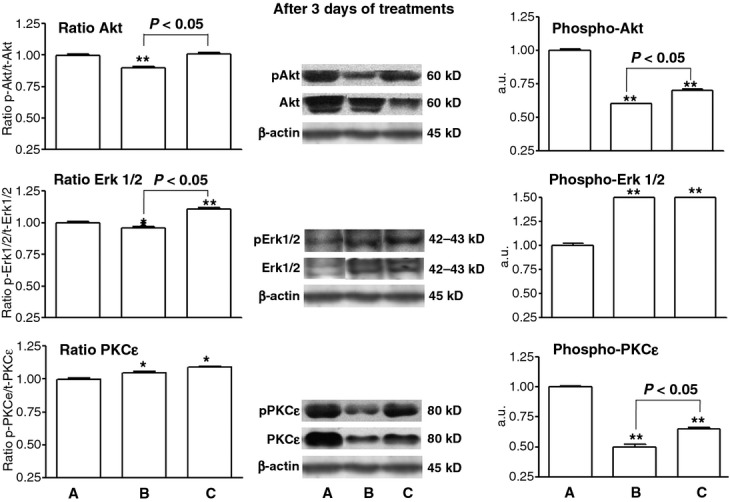
Western blot analysis of Akt (top panels), ERK1/2 (middle panels), and PKCε (bottom panels) after 3-days. (**A**) MSC control group (**B**) MSC treated with FN-PAM-VEGF, and (**C**) MSC treated with free-VEGF-A. The panels on the left are normalized phospho/total kinase ratios. The central panels are representative bands of total and phospho-kinases (some of the presented bands were not juxtaposed in the original film). The panels on the right show the normalized mean values of phospho-enzyme only. We normalized the expression of total kinases and phospho-kinases for each condition to its matched loading control β actin and then where normalized with respect to the mean values of MSCs kept under standard conditions for 3-days (MSC-3). It can be appreciated a reduced Akt phospho/total ratio in FN-PAM-VEGF, an increased ERK1/2 phospho/total ratio in VEGF-A, and an increased phospho/total ratio of PKCε in both FN-PAM-VEGF and VEGF-A groups. *P < 0.05 respect to MSC control group; **P < 0.001 respect to MSC control group. *n* = 4 for each condition.

As can be seen in Figure 7, after 30-min. treatment with VEGF-A there was an increase in Akt, ERK 1/2 and PKCε phosphorylation [24, 25]. On the contrary, FN-PAM-VEGF, which slowly releases VEGF-A (see Fig. 3), does not influence kinase phosphorylation.

After 1 day treatment (Fig. 8), kinase phosphorylations observed at 30-min. in VEGF-A group are no longer present. Moreover, FN-PAM-VEGF induces a significant reduction in phospho-Akt levels. Starting from different levels of total kinases, after 3-days (Fig. 9) the phospho-Akt and phospho-PKCε abundances were still reduced, but more with FN-PAM-VEGF than with free VEGF-A. In particular, the analysis for Akt (Top Panels) evidenced a reduced phospho/total ratio of Akt in FN-PAM-VEGF and a preserved ratio in VEGF-A only. The level of phospho-Akt was lower in treated groups (both VEGF-A and FN-PAM-VEGF). Importantly, phospho-Akt level was higher in VEGF-A than in FN-PAM-VEGF group. Also the analysis for PKCε (Bottom Panels) evidenced an increased phospho/total ratio in both FN-PAM-VEGF and VEGF-A; however phospho-PKCε level resulted lower than the MSC group, in both FN-PAM-VEGF and VEGF-A. Yet the phospho-PKCε level was higher in VEGF-A than FN-PAM-VEGF group. On the contrary, after treatments phospho-ERK 1/2 levels were greatly and similarly increased in both VEGF-A and FN-PAM-VEGF groups (Middle Panels). The analysis of ERK1/2 showed an increase for phospho/total ratio in VEGF group only.

**Fig. 10:**
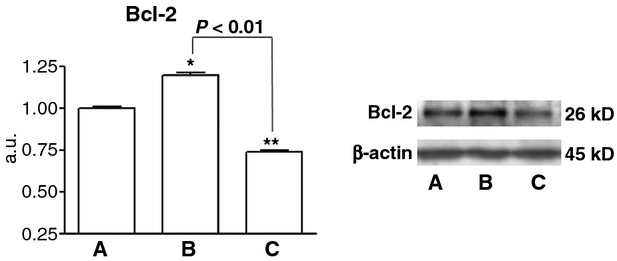
Representative blots and mean levels of the anti-apoptotic factor, Bcl-2 after 3-days treatment with active factors (free-VEGF-A and FN-PAM-VEGF). (A) MSC control group (B) MSC treated with FN-PAM-VEGF, and (C) MSC treated with free-VEGF-A. We normalized the expression of Bcl-2 for each condition to its matched loading control β actin and then where normalized with respect to the mean values of MSC-3. It can be appreciated an increase of Bcl-2 in FN-PAM-VEGF and a decrease in VEGF-A group. *P < 0.05 respect to MSC-3; **P < 0.001 respect to MSC-3. *n* = 4 for each condition.

Figure 10 shows that after 3-days of treatment, Bcl-2, a marker of anti-apoptotic activity involved in the survival mechanisms, was preserved by FN-PAM-VEGF and decreased by free VEGF-A treatment.

#### Analysis after H/R

**Fig. 11:**
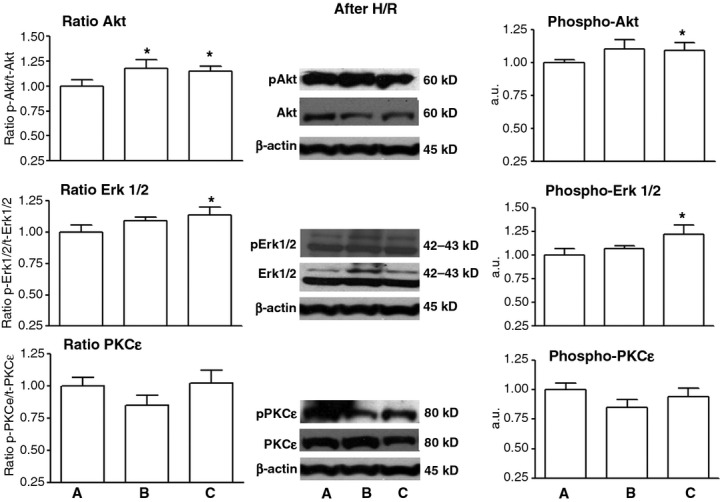
Western blot analysis of Akt (top panels), ERK1/2 (middle panels), and PKCε (bottom panels) after hypoxia/reoxygenation (H/R). (**A**) MSC control group (**B**) MSC treated with FN-PAM-VEGF, and (**C**) MSC treated with free-VEGF-A. The panels on the left are normalized phospho/total kinase ratios. The central panels are representative bands of total and phospho-kinases. The panels on the right show the normalized mean values of phospho-enzyme only. We normalized the expression of total kinases and phospho-kinases for each condition to its matched loading control β actin and then where normalized with respect to the mean values of MSCs subjected to H/R. It can be appreciated an increased Akt phospho/total ratio in both FN-PAM-VEGF and VEGF-A groups, an increased ERK1/2 phospho/total ratio in VEGF-A only, and an unchanged phospho/total ratio of PKCε in both FN-PAM-VEGF and VEGF-A groups. *P < 0.05 respect to MSC control group; *n* = 3 for each condition.

**Fig. 12:**
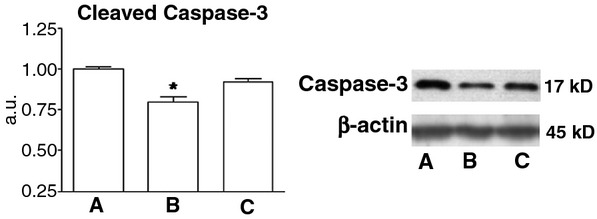
Representative blots and mean levels of the apoptotic factor, cleaved Caspase-3 after hypoxia/reoxygenation (H/R). (A) MSC control group (B) MSC treated with FN-PAM-VEGF and (C) MSC treated with free-VEGF-A. We normalized the expression of cleaved Caspase-3 for each condition to its matched loading control β actin and then where normalized with respect to the mean values of MSC. It can be appreciated a decrease of cleaved Caspase levels in FN-PAM-VEGF group only. *P < 0.05 respect to MSC-3. *n* = 3 for each condition.

Figures 11 and 12 show the studied markers after H/R. Also in these figures (A) MSC control group, (B) MSC treated with FN-PAM-VEGF, and (C) MSC treated with free-VEGF-A.

Starting from different levels of total kinases, after H/R phospho-Akt and phospho-ERK 1/2 levels were greatly and similarly increased in VEGF-A group only, whereas phospho-PKCε abundances were unchanged by treatment with VEGF-A or FN-PAM-VEGF (Fig. 1). In particular, the analysis for Akt (Top Panels) evidenced a increased phospho/total ratio of Akt in FN-PAM-VEGF and in VEGF-A groups. The analysis of ERK1/2 (Middle Panels) showed an increase for phospho/total ratio in VEGF-A group only. The analysis for PKCε (Bottom Panels) evidenced an unchanged phospho/total ratio in both FN-PAM-VEGF and VEGF-A.

Figure 2 shows that after H/R, cleaved Caspase-3, a marker of apoptosis, was significantly lower in FN-PAM-VEGF group.

## Discussion

The major new finding in this study is that sustained VEGF-A release by FN-PAM-VEGF induces a greater resistance of MSCs to H/R, whereas free VEGF-A induces a greater increase in MSC proliferation in normoxia.

Accordingly Akt and PKCε are more phosphorylated/activated by free VEGF-A than by FN-PAM-VEGF, whereas MSCs treated with FN-PAM-VEGF show a higher level of the anti-apoptotic factor Bcl-2 with respect to VEGF-A only. Although PKCε may induce Bcl-2 biosynthesis, the most important action of this kinase is to stimulate proliferation. The increased amount of Bcl-2 due to FN-PAM-VEGF could also be attributed to the presence of fibronectin which is known to promote cell survival *via* multiple effects, including Bcl-2 over-expression [42].

Our results on proliferative mediators and anti-apoptotic agents corroborate the different effects of the two modalities of VEGF-A application (free VEGF-A *versus* FN-PAM-VEGF) on cell proliferation in normoxia and cell survival in hypoxia. Difference may be due to different timing of exposure to VEGF-A and kinases responses. In fact, while both treatment and pre-treatment with free VEGF-A increase cell proliferation more than FN-PAM-VEGF in normoxia, only pre-treatment with FN-PAM-VEGF limits cell mortality after H/R. Since pro-proliferative kinases were more rapidly activated by free VEGF-A than FN-PAM-VEGF, we observe an enhanced proliferation with free VEGF-A. Moreover, after 3 days of treatment the levels of phospho-ERK 1/2 – involved in the survival mechanisms – are similarly increased by both treatments (free VEGF-A and FN-PAM-VEGF), and the anti-apoptotic activity (*e.g*. Bcl-2) is better preserved by FN-PAM-VEGF than free VEGF-A; thus, we suggest that these factors may be responsible of the greater resistance to hypoxia challenging by MSCs pre-treated with FN-PAM-VEGF.

Although some of the studied factors may exert both pro-proliferative and anti-apoptotic effects (*e.g*. phospho-Akt), we can observe that Blc-2, a specific anti-apoptotic agent, is increased by FN-PAM-VEGF, whereas agents with proliferative properties, such as phospho-Akt and phospho-PKCε, are higher when VEGF-A is given alone. The phospho-ERK 1/2 is similarly increased by both treatments; whereas ERK 1/2 shows an increase in phospho/total ratio in VEGF-A group only.

These results suggest a stronger anti-apoptotic activity by FN-PAM-VEGF with respect to VEGF-A alone. Yet VEGF-A stimulates mainly proliferative agents. Therefore in normoxia the cell number is increased greatly by VEGF-A; whereas in H/R cell death is mainly limited by FN-PAM-VEGF. Moreover, as said, we should consider that although Akt is both a proliferative and an anti-apoptotic agent, Bcl-2 is an anti-apoptotic factor specifically activated against H/R [43]. In fact, after H/R in FN-PAM-VEGF group phospho-Akt/Akt ratio is increased and cleaved Caspase-3 is reduced. Therefore, it is likely that VEGF-A alone promotes proliferation in normoxia (*via* Akt phosphorylation) thus increasing the number of viable cells. Yet FN-PAM-VEGF increases cell survival by reducing apoptosis (*via* Bcl-2 preservation in normoxia, as well as *via* Akt ratio increase and cleaved Caspase-3 reduction after H/R).

The efficiency of the transplanted MSCs is limited by several factors, including a hostile environment such as that occurring in inflammation, reoxygenated or aging tissue and the lack of growth factors [7]. Several approaches have been suggested to overcome these hurdles. Strategies using pre-treated cells with MSC co-culture or co-transplantation with other cells have been investigated to improve cell survival in hypoxic conditions. Some studies have suggested that endothelial cells would participate to cell survival, proliferation as well as vascularization in such conditions [44–46]. For example, this effect may be related to paracrine activity of HUVEC on MSCs, as recently reported *in vitro* [44, 47]. A dual cell transplantation of HUVEC with MSCs has been tested also *in vivo*, with an improvement of the differentiation of MSCs in smooth muscle cells and with subsequent improvement of vasculogenesis and post-ischemic cardiac function [46, 48]. However, studies have on the contrary reported the paracrine effect of MSCs on endothelial cells *in vitro* under hypoxic condition [45, 49] but also the effect of the contemporary transplantation of MSCs and endothelial cells *in vivo* using ischemic models [46, 48, 50]. Indeed, the secretion of different cytokines by MSCs is well known, and among them VEGFs are well documented [22]. Despite these beneficial effects, VEGFs produced by cells is not enough to promote a sufficient protection to the transplanted MSCs; therefore *in situ* delivery of exogenous VEGFs has been proposed to improve cell survival [23, 51, 52].

Another feasible approach is the development of biopolymer-based growth factor delivery for tissue repair, which may be beneficial even in the case of reduced availability of stem cell to implant. Several kinds of scaffolds composed of synthetic or natural polymers have been tested with different experimental models in preclinical and clinical studies. PAMs have been developed which combine, in an adaptable and simple device, *in situ* controlled drug delivery and implantation of cell adhered onto biomaterials-based scaffolds. The PAMs used in the present study are biocompatible and biodegradable microspheres made of poly (D,L-lactide-co-glycolide acid (PLGA)) with a biomimetic surface of ECM molecules supplying a three-dimensional structure for the cell both *in vitro* and *in vivo* after transplantation. They may also be engineered to release a therapeutic factor in prorogated manner [53]. Therefore these combined parameters may promote or maintain cell survival, differentiation and integration in the host tissue after complete degradation of the carrier. In particular, adhesion to structural glycoproteins of the ECM seems to be necessary for cell survival and fibronectin is considered as a protective factor for many adherent cells [54]. Moreover, it has been recently suggested a novel pro-survival pathway involving integrin receptors and proteins of Bcl-2 family which can be stimulated by fibronectin [42]. Thus, we cannot rule out that the differences observed between VEGF-A and FN-PAM-VEGF groups inherent their effects on MSC survival can be due, at least in part, to the interaction of VEGF-A with fibronectin.

VEGF-A production is a crucial component of stem cell-mediated cardioprotection as evidenced by a reduction in post-ischemic myocardial functional recovery following intracoronary infusion of MSCs with targeted VEGF-A suppression using siRNA [51]. Here using either VEGF-A alone or in combination with FN-PAMs (FN-PAM-VEGF) we observed an enhanced proliferation of MSCs in normoxia. However, VEGF-A is more effective than FN-PAM-VEGF in inducing proliferation. On the other hand, in hypoxic conditions FN-PAM-VEGF was more protective than VEGF-A against H/R challenging. Differences between free VEGF-A and FN-PAM-VEGF may be due also to the VEGF-A kinetics of release elicited by the FN-PAM (see Fig. 3). Since it has been underlined that reduced proliferation might favor stem cell differentiation, whereas highly proliferating stem cells hardly differentiate [55, 56], we suggest that functionalized FN-PAM inducing a limited proliferation and an increased resistance to hypoxia/reoxygenation may be a novel approach for enhancing MSC survival and differentiation and, thus, regeneration of damaged tissue. For instance, FN-PAM-VEGF can be used when the cell loss affects the heart, to limit heart failure [57]. In fact MSCs can differentiate into vascular endothelial cells and cardiomyocytes and can improve heart function even *via* vascular paracrine signaling effects [3, 4, 58, 59].

Although here we do not report data about the effects of PAM and/or VEGF-A for periods longer than a week or *in vivo*, toxicity and lack of effectiveness are not of concern. In fact, the angiogenic properties of VEGF-A slowly released by microspheres have been demonstrated to promote localized neovascularization at the subcutaneous site in the rat [*e.g*. [29]. Moreover, our group has confirmed in experimental studies both the ability of FN-PAM-VEGF to stimulate the functionality of endothelial progenitor cells *in vitro* [60] and the biocompatibility or lack of toxicity of PAM in the brain *in vivo* [27, 61]. Yet, these PLGA microspheres, which are biodegradable polymer fully metabolized by the organism, have been used in clinical trials for treating gliomas [61]. Finally, PLGA implantable devices are approved by the FDA and PLGA sutures are widely used in surgery.

## Conclusions

Our data suggest that *in vitro* treatment with free VEGF-A enhances MSC proliferation, most likely trough the rapid activation of pro-survival kinases and persistence of ERK 1/2 pathway. Yet, controlled release of VEGF-A from FN-PAM-VEGF limits mainly MSC death *via* both ERK 1/2 and Bcl-2 mechanisms, thus enhancing MSC survival in post-hypoxic environment.

Our data are in line with the viewpoint that in therapies with cell transplantation are needed approaches adapted to the varying conditions. We suggest that the use of FN-PAM complexed with growth factors could be considered a new approach to improve MSC survival in hostile environments such as inflammatory and post-ischemic tissue. If confirmed in an appropriate pre-clinical model, this approach may be envisaged for future clinical studies on cardiac tissue regeneration.
